# Long-Term SARS-CoV-2 Antibody Seroprevalence in Blood Donors, Italy

**DOI:** 10.3201/eid2907.221745

**Published:** 2023-07

**Authors:** Martina Ferrari, Lorenza Di Marco, Alessandra Pivetti, Stefania Paduano, Chiara Vecchi, Veronica Bernabucci, Rosina Maria Critelli, Simone Lasagni, Monica De Maria, Donatella Venturelli, Monica Pecorari, Giorgia Boaretto, Giulia Fregni Serpini, Dante Romagnoli, Roberto Mantovani, Giovanni Battista Ceccherelli, Erica Villa

**Affiliations:** University of Modena and Reggio Emilia and Azienda Ospedaliero-Universitaria di Modena, Modena, Italy (M. Ferrari, L. Di Marco, A. Pivetti, V. Bernabucci, R.M. Critelli, S. Lasagni, E. Villa);; Clinical and Experimental Medicine PhD Program, University of Modena and Reggio Emilia, Modena (L. Di Marco, S. Lasagni);; University of Modena and Reggio Emilia Modena (S. Paduano);; Azienda Ospedaliero-Universitaria di Modena, Modena (C. Vecchi, M. De Maria, D. Venturelli, M. Pecorari, G. Boaretto, G.F. Serpini, D. Romagnoli, G.B. Ceccherelli);; Associazione Italiana Volontari Sangue, Modena (R. Mantovani)

**Keywords:** COVID-19, SARS-CoV-2, antibodies, blood donor population, serologic analysis, Italy, viruses, respiratory infections

## Abstract

We evaluated SARS-CoV-2 antibody response in voluntary blood donors in Italy at different timepoints. Immediately after lockdown easing, 908/25,657 donors (3.5%) had low IgG titers against nucleocapsid. In the next 2 years, titers increased despite few COVID-19 symptoms. On multivariate analysis, allergic rhinitis was associated with reduced risk for symptomatic COVID-19.

Antibodies against different viral epitopes develop in persons infected with SARS-CoV-2 (*1*,*2*). We evaluated SARS-CoV-2 antibody levels and types in a voluntary blood donor (VBD) population in Modena, Italy, at different timepoints from the beginning of the COVID-19 pandemic and examined the effects of clinical and biologic factors, including natural and vaccine-associated SARS-CoV-2 antibody presence, on antibody development and clinical outcomes.

During July–December 2020, a total of 908/25,657 (3.5%) sequential VBDs whose donations were positive for SARS-CoV-2 nucleocapsid IgG were referred to our hospital for clinical evaluation and oronasopharyngeal molecular swab testing. All but 4 (0.4%) were negative for spike IgM. We repeated serologic and swab testing in the same donors after 3 months and 22 months (Figure; Appendix Table 1). Apart from rhinitis (n = 68, 7.5%), obesity (n = 60, 6.6%), and hypertension (n = 98, 10.8%, all in patients taking angiotensin-converting enzyme [ACE] inhibitors), the VBDs were healthy. Symptomatic COVID-19 infection occurred in 9 (9.1%) ACE users and 221 (31.0%) non–ACE users (p<0.001 by χ^2^ test).

**Figure:**
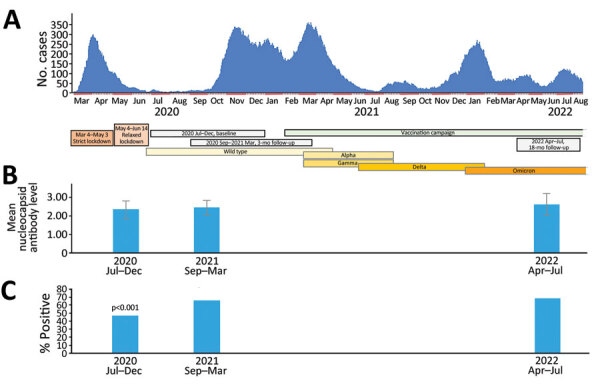
Study timepoints plotted against hospital admissions for COVID-19 in study of SARS-CoV-2 seroprevalence among voluntary blood donors in Modena, Italy. A) After the first wave of COVID-19 in March 2020–April 2020, which followed 2-month strict and 1-month relaxed lockdown periods, a 4-month period (June 2020–September 2020) of almost no hospital admissions associated with low (3.5%) SARS-CoV-2 antibody seroprevalence ensued. Thereafter, a sequence of intercurrent waves occurred with only a very short period of few admissions in July 2021. Titers of antibodies against the virus nucleocapsid (tested in the same donors at baseline and after 3 and 22 months) increased throughout the observation period although not significantly. Colored horizontal bars indicate prevalent variants throughout the observation periods. B) Mean antibody titers against nucleocapsid among strongly positive donors were significantly higher at the 2nd and 3rd testing points compared with baseline. Error bars indicate standard deviation. C) Percentages of donors with strongly positive antibody response against the nucleocapsid, tested on the whole cohort. p value indicates the comparison of percentages at 2nd and 3rd testing points to percentage at baseline.

Of the 908 VBDs, 208 (22.9%) reported histories of symptomatic COVID-19 infection in the 3 months before their donation. Apart from fever (5.2%) and asthenia (2.4%), signs and symptoms at initial, 3-month, and 22-month follow-up assessments were minor. Of the 908 VBDs who tested positive for nucleocapsid SARS-CoV-2 IgG at baseline, 27 (2.9%) were also positive by oronasopharyngeal swab test. When tested again 3 months later, 33/908 (3.6%) had a positive oronasopharyngeal swab test. One VBD was positive on both occasions. Very low viral load prevented SARS-CoV-2 subtype lineage identification. No VBD required hospitalization during the study period. Most VBDs received SARS-CoV-2 vaccines beginning in February 2021; 54 (5.9%) did not. Vaccinated VBDs had significantly lower titers of nucleocapsid IgG and significantly higher titers of IgG against the spike protein receptor binding domain and of neutralizing antibodies, compared with the unvaccinated VBDs (Appendix Figure 1).

Titers of SARS-CoV-2 antibody types within vaccinated or unvaccinated VBDs in 2022 were not related to VBDs’ prevaccination histories of symptomatic COVID-19. Titer of antibodies against the nucleocapsid was significantly higher in symptomatic cases compared with those who were not symptomatic in 2022; this observation was consistent in both vaccinated and unvaccinated donors (p<0.001 by t-test) (Appendix Table 3). Logistic regression revealed that allergic rhinitis was associated independently with a reduced risk for symptomatic COVID-19 (Table).

**Table:** Factors associated with the risk of severe COVID-19 development in blood donors, Italy*

	Univariate analysis			Multivariate analysis	
Variable	OR (95% CI)	p value		OR (95% CI)	p value
Age	0.985 (0.973–0.996)	**0.008**		0.984 (0.964–1.005)	0.135
Sex	1.047 (0.755–1.453)	0.783			
Body mass index	1.001 (1.000–1.002)	0.178			
Smoking	0.498 (0.193–1.287)	0.150			
Concurrent conditions	0.600 (0.299–1.205)	0.151			
Allergic rhinitis	0.180 (0.043–0.757)	**0.019**		0.170 (0.40-0.719)	**0.016**
ACE inhibitor use	0.181 (0.089–0.365)	**<0.011**		1.036 (0.452–2.373)	0.933
Chronic therapies	0.793 (0.392–1.6020)	0.517			
Influenza vaccination	0.678 (0.276–1.666)	0.397			
Family case of SARS-CoV-2 infection	0.938 (0.407–2.159)	0.880			
Education level	1.248 (0.824–1.892)	0.296			
Municipality of residence	1.011 (0.996–1.027)	0.134			

*Bold text indicates significance. ACE, angiotensin-converting enzyme.

This 2-year prospective study showed that SARS-CoV-2 seroprevalence in VBDs is a reliable indicator of the epidemiologic situation in the general population. The very low initial percentage (3.5%) of VBDs with SARS-CoV-2 nucleocapsid antibodies mirrored that in the general population in the same province (2.5%) (*3*); the percentage of antibodies was an indicator of natural infection. Nucleocapsid IgG levels were significantly higher (5%–6.4%) in VBDs in nearby regions tested 3 months earlier than ours, reflecting much greater SARS-CoV-2 exposure because of a relaxed containment policy. The policy implemented in the study area was extremely strict, resulting in almost no hospital admission for COVID-19 by the end of the lockdown period (*4*,*5*). In towns in the study area close to the Lombardy border, 7.8%–18.8% of the general population carried SARS-CoV-2 antibodies (*6*). Given the extremely strict lockdown policy, however, the general population had marginal immunity against SARS-CoV-2, despite good titers in persons testing positive. The infection rate rose with the easing of lockdown, creating an 8-month-long emergency situation for hospitals. The observed stable elevation of titers of antibodies against the nucleocapsid and other epitopes, independent of vaccination, suggests equivalent elevation of viral circulation, as described previously (*7*,*8*), although vaccination reduced COVID-19’s clinical severity and lethality.

SARS-CoV-2 infection symptoms in this large VBD cohort were mild, consistent with previous findings (*9*). Univariate analysis indicated that the VBDs’ young median age and good health (active, with few concurrent conditions) likely contributed to that outcome. The association of allergic rhinitis with a reduced risk for symptomatic COVID-19 could be linked with the reduction of ACE2 receptors in the epithelial cells of inflamed airways of affected VBDs; we could not rule out a connection between treatment for rhinitis and COVID-19 (*10*).

A limitation of the study is the lack of information on the exact timing of the primary infection; symptoms of COVID-19 were rare, and antibodies against nucleocapsid, which have higher waning than those against the spike protein, were less valuable as indicators of infection. However, the results of this long-term follow-up of the VBD population before and after SARS-CoV-2 vaccination offers an interesting perspective of the epidemiologic events associated with SARS-CoV-2 infection, especially the paradoxical effect of strict lockdown.

Appendix Additional information about long-term SARS-CoV-2 antibody seroprevalence in blood donors, Italy.
