# Supplementary material for: Long-Term SARS-CoV-2 Antibody Seroprevalence in Blood Donors, Italy
Source: Emerg Infect Dis. 2023 Jul;29(7):1479–81. doi: 10.3201/eid2907.221745 (PMC10310387; doi:10.3201/eid2907.221745)
Supplement: Appendix — Additional information about long-term SARS-CoV-2 antibody seroprevalence in blood donors, Italy. [file 22-1745-Techapp-s1.pdf]

Article DOI: https://doi.org/10.3201/eid2907.221745

EID cannot ensure accessibility for supplementary materials supplied by authors. Readers who have difficulty accessing supplementary content should contact the authors for assistance.

Long-Term SARS-CoV-2 Antibody Seroprevalence in Blood Donors, Italy

Appendix

Participants

After providing written informed consent, voluntary blood donors (VBDs) were tested for severe acute respiratory syndrome coronavirus 2 (SARS-CoV-2) in July 2020–December 2020 as part of a project promoted by the government of the Regione Emilia-Romagna. The donations, collected by AVIS through the network of sampling centers, were tested on the same day by the Blood Bank. Donors who tested positive were referred within three days to Gastroenterology Unit, where they underwent oro-nasopharyngeal swab and clinical evaluation.

The study protocol was approved by the Area Vasta Emilia Nord ethics committee (535/2020/OSS/AOUMO).

Serologic and Oro-Nasopharyngeal Molecular Swab Testing

Serologic and oro-nasopharyngeal molecular swab testing was first performed in July–December 2020: each VBD was then recalled after 3 months from the initial testing (September 2020–March 2021), and after 22 months (April 2022–July 2022) in order to evaluate the evolution of his/her antibody status. At each visit, the VBDs completed a detailed questionnaire on their clinical health (including chronic diseases and therapies, risk factors for SARS-CoV-2 infection [type of employment, familial cases], and influenza and SARS-CoV-2 vaccination).

Serological Analysis

The blood samples of the VBDs were tested for IgM antibodies against the SARS-CoV-2 spike protein and IgG antibodies against the nucleocapsid structure protein using a chemiluminescent microparticle capture immunoassay (Alinity; Abbott Ireland, Sligo, Ireland). Index values between 1.0 and 1.4 were reported as weakly positive, and >1.4 as positive (Bryan et al., doi: 10.1128/JCM.00941-20). At the third assessment, semiquantitation of IgG antibodies against the receptor binding domain of the virus spike protein using a chemiluminescent microparticle capture immunoassay (results expressed as Arbitrary Units per mL, AU/ml)(SARS-CoV-2 IgG II Quant, Alinity; Abbott, Sligo, Ireland) and of neutralizing antibodies against SARS-CoV-2 (results expressed as Arbitrary Units per mL, AU/mL) (Latex immunoturbidimetric assay; SGM Italia spa, Rome, Italy) were also performed. This inhibition test is based on the principle of blocking the interaction between RBD and ACE2. It cannot detect all neutralizing antibodies, but only antibodies blocking RBD (Lu, Y, Wang, J, Li, Q, Hu, H, Lu, J, Chen, Z. Advances in Neutralization Assays for SARS-CoV-2. Scand J Immunol. 2021; 94: 224– 238. https://doi.org/10.1111/sji.13088).

Molecular Analysis

For the determination of SARS-CoV-2, RNA was extracted starting from UTM-RT transport medium for viruses (Copan, Italia S.p.A), in which the swab had been immersed after the oro-rinopharyngeal or nasopharyngeal sampling. 500ul of this medium was loaded on the Alinity m instrument (Abbott Molecular Inc, Des Plaines, IL, USA) and processed for the determination of SARS-CoV-2 with the Alinity m SARS-CoV-2 AMP Kit and Alinity m kits SARS-CoV-2 CTRL Kit.

The Alinity m SARS-CoV-2 assay is a dual target assay for the RdRp and N genes. An RNA sequence unrelated to the SARS-CoV-2 sequence is introduced into each specimen at the beginning of sample preparation. This unrelated RNA sequence is simultaneously amplified by RT-PCR and serves as an internal control (IC) to verify that the reaction has proceeded correctly.

Statistical Analysis

Dichotomous and continuous variables were analyzed using Fisher's exact test, the χ^2 test, bivariate (Pearson) correlation analysis, paired and unpaired *t*-tests, and nonparametric tests for independent samples (Mann–Whitney *U* test). *P* values <0.05 were considered to be significant. In a univariate analysis, the following variables were examined: age, sex, body mass index, smoking habit, concurrent conditions, allergic rhinitis, angiotensin-converting enzyme inhibitor use, chronic therapies, influenza vaccination, familial cases of SARS-CoV-2, education level, municipality of residence, and occupation conferring risk of SARS-CoV-2 infection. Variables with *p* values <0.10 in the univariate analyses were included in a multivariate model. Logistic regression analysis was performed to identify variables favoring SARS-CoV-2 infection. The statistical analyses were performed using PASW Statistics software (ver. 28; IBM Corporation, Armonk, NY, USA).

Appendix Table 1. Clinical and demographic characteristics of blood donors in study of SARS-CoV-2 antibodies

Characteristic	n (%) or mean ± SD, N = 908
Sex, M	601 (66.2)
Age (median), y	44.5 ± 13.6 (48)
Body mass index (kg/m²)	
M	25.8 ± 3.7
F	24.8 ± 3.9
Smoking (n = 535)	
Never	467 (87.3)
Active	55 (10.3)
Former	13 (2.3)
Concurrent conditions, n = 542	135 (24.9)
Hypertension/heart conditions	98 (10.8)
Obesity	60 (6.6)
Chronic respiratory condition	9 (1.0)
Gastrointestinal condition	2 (0.2)
Other	14 (1.5)
Angiotensin-converting enzyme inhibitor use, n = 722	98 (13.5)
Allergic rhinitis	68 (7.5)
Influenza vaccination	60 (15.2)
SARS-CoV-2 vaccination	854 (94.0)
Educational level	
Compulsory school	127 (14.0)
High-school degree	229 (25.2)
University degree	74 (8.1)
Other	478 (52.6)
Job with high infection risk	123 (30.7)
Remote working	56 (13.1)
Type of work	
Employee, no public contact	94 (21.9)
Employee, public contact	74 (17.2)
Factory worker	71 (16.6)
College student	52 (12.1)
Retired person	29 (6.8)
Unemployed	16 (3.7)
Engineer	11 (2.6)
Health worker	11 (2.6)
Farmer	10 (2.3)

Characteristic	n (%) or mean + SD, N = 908
Teacher	9 (2.1)
Artisan	9 (2.1)
Bartender	8 (1.9)
Restaurateur	8 (1.9)
Executive manager	7 (1.6)
Grocer/baker	5 (1.2)
Consultant	5 (1.2)
Shopkeeper	4 (0,9)
Elder caregiver	2 (0.5)
Firefighter	1 (0.2)
Supermarket employee	1 (0.2)
Funeral home worker	1 (0.2)
Solicitor	1 (0.2)

Appendix Table 2. Distribution of strong IgG reactivity against SARS-CoV-2 nucleocapsid and mean titers at different timepoints

	IgG against virus nucleocapsid	
Period	Strongly positive, no. (%)	Mean titer
July–December 2020	434 (47.8)*, **	2.5 ± 2.1§
September 2020–March 2021	600 (66.0)*	2.6 ± 2.7§
April 2022–July 2022	618 (68.0)**	2.7 ± 2.7§

*, **p<0.001 (Fisher exact test)
§Not significant (t test).

Appendix Table 3. Relationship between SARS-CoV-2 antibodies against the nucleocapsid in blood donors*

	Symptomatic COVID-19		
Blood donors	No	Yes	p value
Vaccinated blood donors	Symptomatic COVID in 2020 (Baseline)		
IgG against nucleocapsid (Index value)	2.4 ± 2.7	2.5 ± 2.7	0.690
IgG against RBD (AU/mL)	15.551 ± 21.885	16.672 ± 32.137	0.835
Neutralizing antibodies (AU/mL) (antibodies blocking RBD)	87.5 ± 8.3	86.8 ± 8.0	0.743
	Symptomatic COVID-19 in 2022		
IgG against nucleocapsid (Index value)	1.9 ± 2.7	4.6 ± 3.1	<0.001
IgG against RBD (AU/mL)	15.427 ± 25.066	16.923 ± 11.888	0.711
Neutralizing antibodies (AU/mL) (antibodies blocking RBD)	87.9 ± 7.8	86.3 ± 9.5	0.743
Unvaccinated blood donors	Symptomatic COVID-19 in 2020 (Baseline)		
IgG against nucleocapsid (Index value)	4.7 ± 2.9	5.6 ± 4.4	0.720
IgG against RBD (AU/mL)	4.506 ± 4.640	1.995 ± 1.762	0.479
Neutralizing antibodies (AU/mL) (antibodies blocking RBD)	68.1 ± 30.9	64.2 ± 17.8	0.870
	Symptomatic COVID-19 in 2022		
IgG against nucleocapsid (Index value)	0.7 ± 0.9	6.1 ± 2.0	<0.001
IgG against RBD (AU/mL)	2.306 ± 3.939	4.663 ± 4.536	0.436
Neutralizing antibodies (AU/mL) (antibodies blocking RBD)	45.1 ± 42.2	77.1 ± 15.3	0.280

*Relationships against the RBD and neutralizing antibodies were tested in vaccinated and unvaccinated VBD stratified by symptomatic COVID-19 in 2020 and in 2022 (Mann-Whitney test). No differences were found for antibodies directed against the RBD or antibodies blocking the RBD. Within each category (vaccinated and unvaccinated) in relation with having had symptomatic COVID in 2020 or 2022, the only significant difference was for antibodies against the nucleocapsid both in vaccinated and unvaccinated VBD who experienced COVID-19 in 2022 (p<0.001 for each category, t test). RBD, receptor-binding domain; VBD, voluntary blood donors.

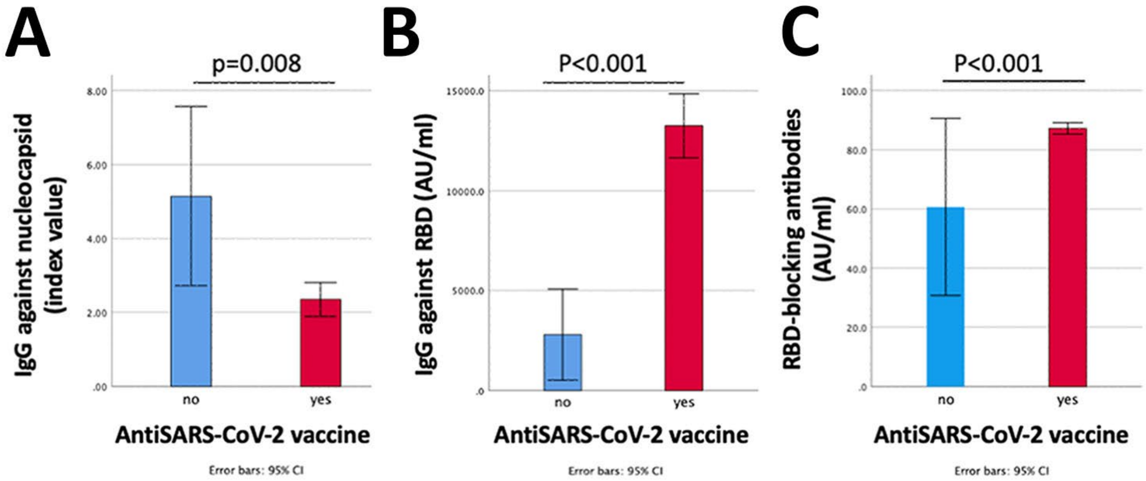

Appendix Figure. Levels of SARS-CoV-2 antibodies in 2022 against the nucleocapsid, against the receptor-binding domain (RBD), and antibodies blocking RBD according to SARS-CoV-2 vaccination status by Mann–Whitney test.
